# Case Report: Dual embolism of the pulmonary and renal arteries after laparoscopy

**DOI:** 10.3389/fmed.2026.1751418

**Published:** 2026-09-10

**Authors:** Zhengguang Yang, Yang Li, Xiao Wang, Bo Xu, Xunhua Xu, Xuanzhe Huang

**Affiliations:** 1 Department of Radiology, China Resources & WISCO General Hospital, Wuhan University of Science and Technology, Wuhan, China; 2 School of Medicine, Wuhan University of Science and Technology, Wuhan, China; 3 Hubei Province Key Laboratory of Occupational Hazard Identification and Control, Wuhan University of Science and Technology, Wuhan, China; 4 Department of Pulmonary and Critical Care Medicine, China Resources & WISCO General Hospital, Wuhan University of Science and Technology, Wuhan, China

**Keywords:** case report, laparoscopic surgery, percutaneous pulmonary artery thrombectomy, pulmonary embolism, renal artery embolism

## Abstract

**Background:**

Concurrent pulmonary and renal artery embolism following laparoscopic surgery is exceedingly rare, yet it may result in severe clinical consequences.

**Case presentation:**

A 78-year-old woman with uterine prolapse underwent laparoscopic-assisted vaginal hysterectomy (LAVH). Shortly after surgery, she developed dyspnea and abdominal discomfort. Computed tomography angiography (CTA) confirmed acute pulmonary embolism accompanied by renal artery embolism. Following a multidisciplinary team (MDT) consultation, she received percutaneous pulmonary artery thrombectomy (PPAT) combined with anticoagulation therapy. Consequently, her symptoms improved markedly, with follow-up imaging demonstrating satisfactory restoration of blood flow in both the pulmonary and renal arteries.

**Conclusion:**

This case represents a rare occurrence of concurrent pulmonary embolism and renal artery embolism following laparoscopic surgery. Effective postoperative venous thromboembolism (VTE) prophylaxis and appropriate follow-up strategies are essential for preventing similar events. Timely diagnosis and MDT management are crucial for optimizing clinical outcomes in such complex cases.

## 1 Introduction

Pulmonary embolism is a common and potentially life-threatening postoperative complication (1). By contrast, concurrent pulmonary and renal artery embolism represents an exceptionally rare clinical entity. The coexistence of pulmonary and renal artery embolism may result in multi-organ dysfunction, profound hemodynamic compromise, and a substantially increased risk of mortality, thereby posing significant diagnostic and therapeutic challenges.

We report a rare case of concurrent pulmonary embolism and renal artery embolism following laparoscopic-assisted vaginal hysterectomy (LAVH). This case demonstrates the rarity and clinical complexity of this dual embolic presentation and suggests a potentially underrecognized association between laparoscopic surgery and thromboembolic events. Furthermore, it underscores the importance of individualized management strategies that can improve outcomes in patients with complex thromboembolic diseases.

## 2 Case description

**Figure 1:**
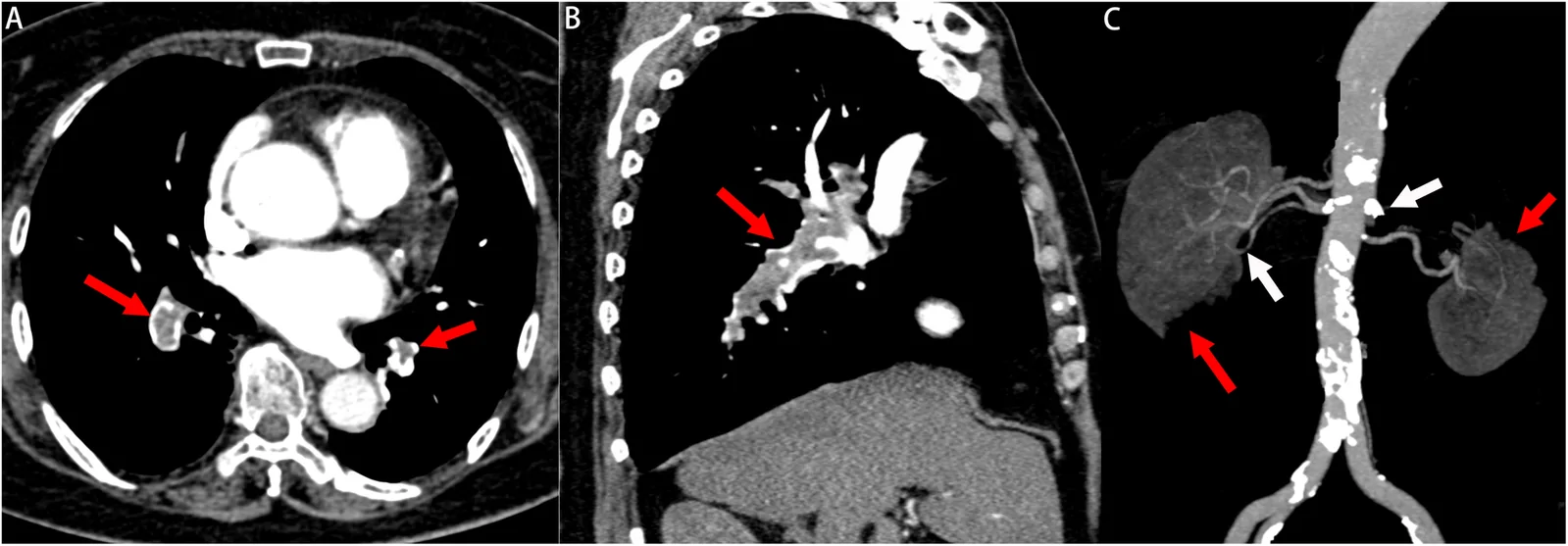
**(A)** axial pulmonary CTA demonstrated multiple intraluminal filling defects in the bilateral lower lobe pulmonary arteries (red arrows), consistent with acute pulmonary emboli. Markedly decreased peripheral pulmonary vascular opacification and sparse distal vascular branches were observed, indicating impaired pulmonary perfusion. **(B)** Multiplanar oblique sagittal reconstruction of pulmonary artery CTA showed extensive thrombotic filling defects involving the right main pulmonary artery and right lower lobar pulmonary artery (red arrows), with extension into segmental branches. **(C)** Mesenteric CTA revealed bilateral accessory renal arteries, with embolism of the left main renal artery and the right accessory renal artery (white arrows). Corresponding wedge-shaped perfusion defects were identified in the mid-to-upper pole of the left kidney and the lower pole of the right kidney (red arrows).

A 78-year-old female patient was admitted with a 1-week history of cough, wheezing, left-sided abdominal distension and pain, and postprandial vomiting. Sixteen days prior to admission, the patient underwent LAVH for uterine prolapse, with an operative duration of 3 h and 45 min. Postoperatively, prophylactic anticoagulation with low-molecular-weight heparin was administered subcutaneously at a dose of 4,000 IU once daily. The patient was discharged on postoperative day 6 after LAVH. At discharge, rivaroxaban 10 mg once daily was prescribed, and early ambulation was recommended. On this admission, the patient presented with cough, wheezing, acute respiratory failure, left-sided abdominal distension and pain, and postprandial vomiting, with a blood pressure of 115/70 mmHg, a heart rate of 94 beats/min, and an oxygen saturation (SpO₂) of 82% (reference range, 95%–100%) on room air. Laboratory findings included a partial pressure of arterial oxygen (PaO₂) of 57.1 mmHg (80–100 mmHg), D-dimer of 8.70 mg/L (0–0.55 mg/L), B-type natriuretic peptide (BNP) of 592.23 pg/mL (0–100 pg/mL), and creatinine of 78.8 μmol/L (44–97 μmol/L). Lower extremity color Doppler ultrasound demonstrated multiple intermuscular venous thromboses in both calves. Echocardiography revealed borderline thickening of the interventricular septum and impaired left ventricular diastolic function. Twenty-four-hour Holter monitoring revealed sinus rhythm with occasional premature atrial contractions, occasional atrial couplets, short runs of atrial tachycardia, frequent premature ventricular contractions, and occasional ventricular trigeminy. Further inquiry revealed that the patient did not take any oral anticoagulants after discharge following LAVH and remained largely bedridden because of fear of postoperative pain, with extremely limited physical activity. Based on the patient's clinical manifestations, both pulmonary embolism and mesenteric ischemia needed to be excluded. Therefore, pulmonary computed tomography angiography (CTA) and mesenteric CTA were performed. Pulmonary CTA demonstrated multiple emboli in the bilateral main and branch pulmonary arteries (Figures 1A,B), accompanied by pulmonary hypertension, as evidenced by a main pulmonary artery diameter of 3.8 cm. Mesenteric CTA revealed no abnormalities in the mesenteric arteries but incidentally detected bilateral accessory renal arteries, calcified plaques at the origins of the renal arteries, and embolisms in the left main renal artery and the right accessory renal artery. Multiple renal infarcts were identified in the mid-to-upper pole of the left kidney and the lower pole of the right kidney (Figure 1C).

## 3 Diagnostic assessment

**Figure 2:**
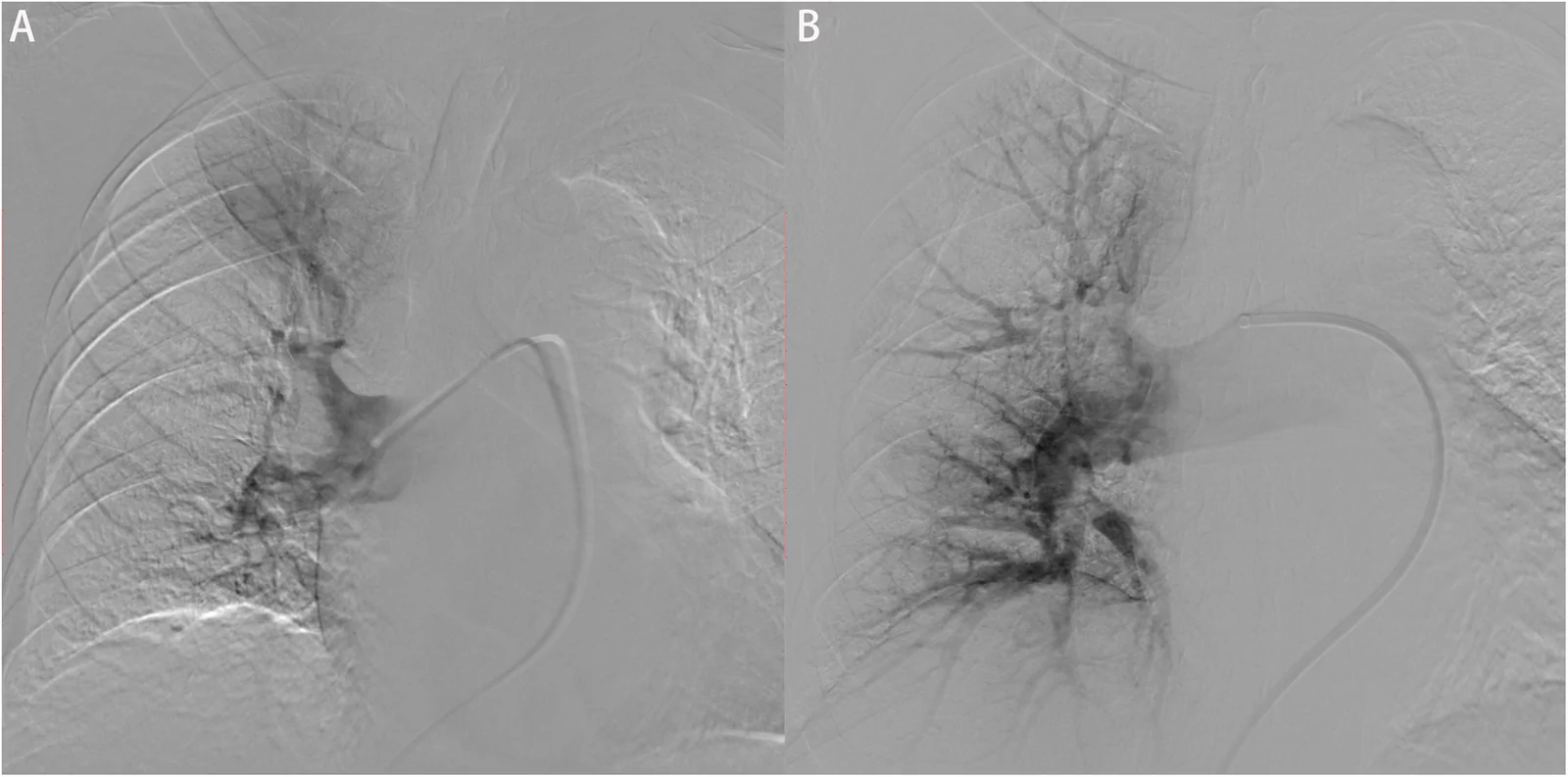
**(A)** Pre-intervention digital subtraction angiography demonstrated a large intraluminal filling defect in the right middle and lower lobar pulmonary arteries, with markedly reduced distal contrast opacification, consistent with acute pulmonary thromboembolism. **(B)** Post-intervention DSA after aspiration thrombectomy showed substantial thrombus removal with restoration of contrast filling in the right middle and lower lobar pulmonary arteries and improved visualization of distal pulmonary vascular branches, indicating successful reperfusion.

**Figure 3:**
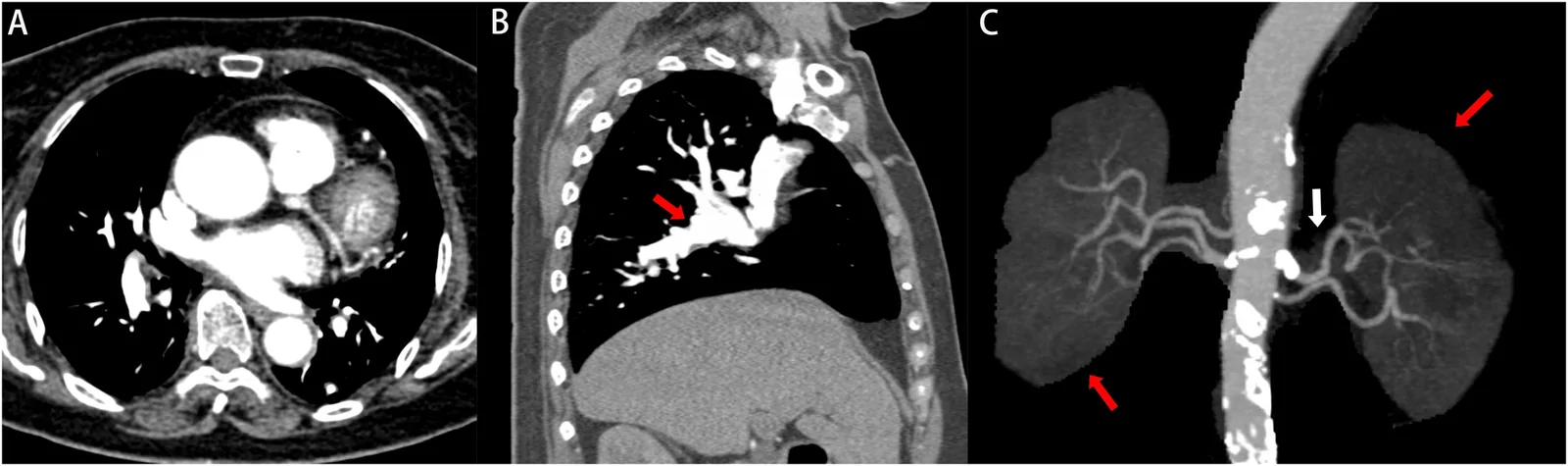
**(A)** pulmonary CTA showed complete resolution of emboli in the bilateral lower lobe pulmonary arteries, with restoration of contrast opacification and improved visualization of distal segmental pulmonary arterial branches. **(B)** Oblique sagittal reconstruction of pulmonary CTA confirmed successful thrombus clearance and restoration of patency in the right pulmonary artery (red arrow). **(C)** Mesenteric CTA demonstrated spontaneous recanalization of the previously embolized left main renal artery (white arrow) and the right accessory renal artery, along with markedly improved renal parenchymal perfusion and near-complete resolution of bilateral renal infarct areas (red arrows).

**Figure 4:**
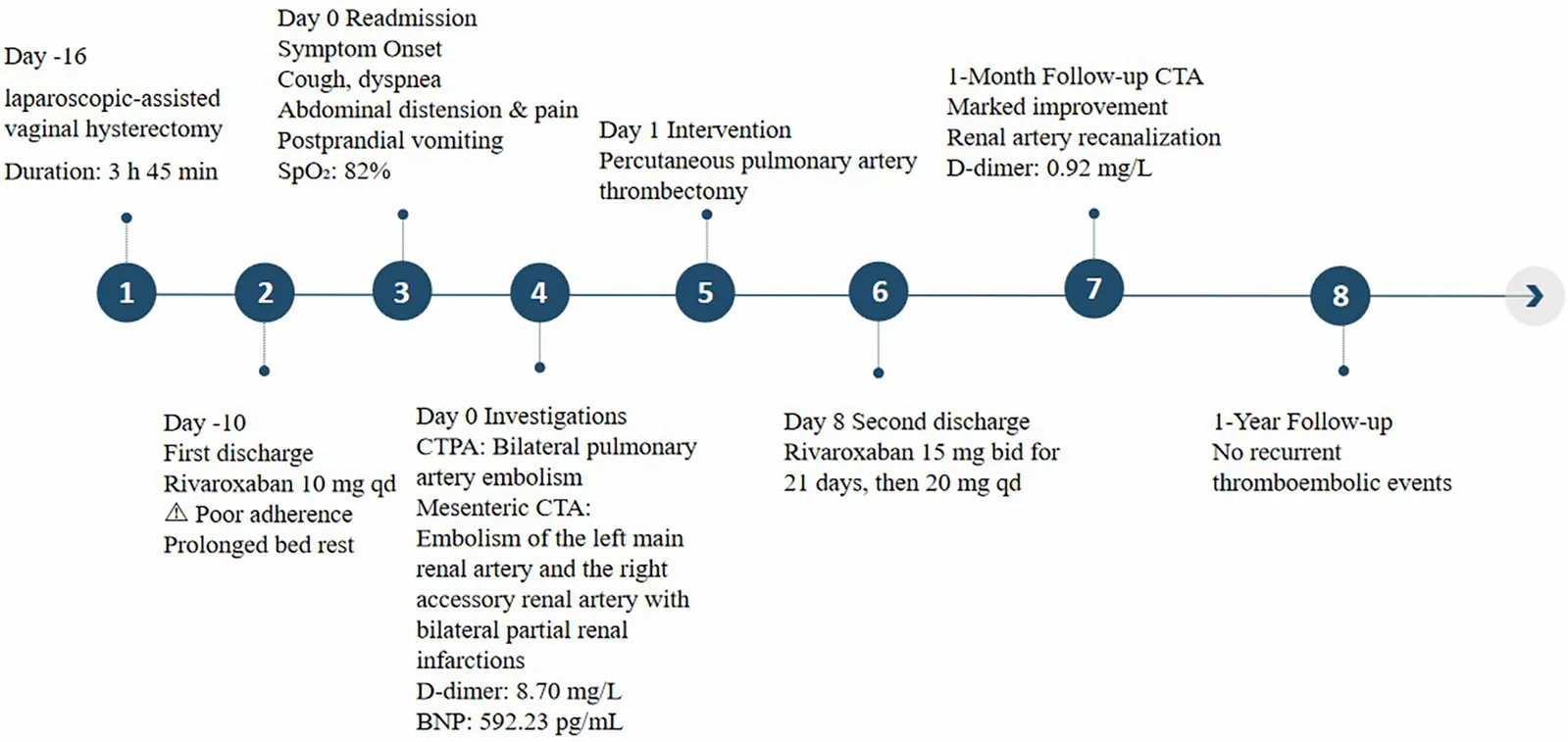
Treatment history of the case.

Following multidisciplinary consultation involving specialists from Respiratory and Critical Care Medicine, Urology, Cardiovascular Medicine, and Interventional Radiology, a diagnosis of acute pulmonary embolism [simplified Pulmonary Embolism Severity Index (sPESI) score: 1] complicated by bilateral renal artery embolism was established. The multidisciplinary team (MDT) considered the patient's condition to be critical because of respiratory failure and pulmonary hypertension. In contrast, renal function remained within the normal range; the kidneys received collateral perfusion through bilateral accessory renal arteries; and the extent of renal infarction was relatively limited. Therefore, a treatment strategy consisting of percutaneous pulmonary artery thrombectomy (PPAT) combined with anticoagulation therapy was adopted. Emergency aspiration thrombectomy was performed using the AcoStream peripheral aspiration catheter system, achieving the successful removal of pulmonary arterial thrombi (Figures 2A,B). Following the procedure, rivaroxaban 15 mg was administered orally twice daily. The patient remained hemodynamically stable, and oxygenation improved progressively. She was discharged on postoperative day 8 following PPAT. After discharge, rivaroxaban 15 mg twice daily was continued for 21 days, followed by maintenance therapy with 20 mg once daily. At the 1-month follow-up, laboratory evaluation demonstrated a reduction in D-dimer levels to 0.92 mg/L (0–0.55 mg/L), while serum creatinine remained within the normal range at 71.4 μmol/L (44–97 μmol/L). Follow-up CTA demonstrated complete resolution of the pulmonary emboli (Figures 3A,B), improvement of pulmonary hypertension, as evidenced by a reduction in the main pulmonary artery diameter from 3.8 cm to 3.4 cm, recanalization of the left main renal artery and the right accessory renal artery, and marked improvement of the bilateral renal infarction lesions (Figure 3C). No recurrent thromboembolic events occurred during the one-year follow-up period. The detailed illustration of the entire treatment process is provided (Figure 4).

## 4 Discussion

### 4.1 Overview of dual embolism and literature context

Concurrent pulmonary embolism and renal artery embolism represent an exceedingly rare clinical entity. In the postoperative setting, symptoms such as dyspnea, chest discomfort, abdominal pain, and flank pain may be mistakenly attributed to routine postoperative recovery rather than underlying vascular occlusion, potentially resulting in delayed diagnosis and treatment (2). Furthermore, the coexistence of embolic events involving multiple vascular territories requires a broader diagnostic perspective than isolated pulmonary or abdominal pathology.

To better characterize the rarity of this condition, a comprehensive literature search was conducted in PubMed and Web of Science covering the period from June 2000 to June 2026. The search strategy combined the keywords (“laparoscopic hysterectomy” OR “laparoscopic-assisted vaginal hysterectomy”) AND (“pulmonary embolism” OR “pulmonary thromboembolism”) AND (“renal artery embolism” OR “renal artery thrombosis”) and was limited to English-language publications with full-text availability. No previously published reports describing concurrent pulmonary embolism and renal artery embolism following LAVH were identified. To our knowledge, this represents a rare case of concurrent pulmonary embolism and bilateral renal artery embolism following laparoscopic surgery.

### 4.2 Pathophysiology of pulmonary embolism in this case

The development of pulmonary embolism in this patient can largely be explained by Virchow's triad of venous stasis, endothelial injury, and hypercoagulability (3). Several perioperative factors likely contributed to thrombus formation. First, noncompliance with postoperative oral anticoagulation therapy and prolonged immobilization likely contributed to an increased risk of deep vein thrombosis. Second, laparoscopic hysterectomy required prolonged operative time and pneumoperitoneum, both of which may impair venous return from the lower extremities and pelvis. Third, surgical trauma and anesthesia can activate coagulation pathways and promote a transient hypercoagulable state. The presence of bilateral calf intermuscular venous thrombosis on ultrasonography strongly supported a venous thromboembolic origin of pulmonary embolism in this patient. These findings are consistent with previous studies demonstrating that prolonged operative duration and postoperative immobility are independent risk factors for venous thromboembolism after gynecological surgery (4).

### 4.3 Pathophysiology of renal artery embolism in this case

The mechanism underlying renal artery embolism in this patient was more complex. First, the patient's failure to adhere to prescribed anticoagulation therapy after discharge, together with prolonged postoperative immobilization due to fear of pain, was likely one of the most important contributing factors to embolism. Second, the transient hypercoagulable state induced by surgery may have further increased the thrombotic susceptibility (5, 6). In addition, the presence of bilateral accessory renal arteries and relatively small main renal arterial trunks may have altered local hemodynamics, potentially contributing to flow disturbances and *in situ* thrombosis (7). Finally, calcified atherosclerotic plaques at the origins of both renal arteries may have provided an additional substrate for embolism (8). These factors may have acted synergistically to increase the likelihood of renal artery embolism.

Several alternative embolic mechanisms were also considered. Transthoracic echocardiography did not demonstrate evidence of an intracardiac shunt. Furthermore, paradoxical embolism most commonly involves the cerebral or peripheral systemic arteries. In the present case, isolated bilateral renal artery embolism occurred in the absence of clinical manifestations suggestive of embolic involvement in other common systemic arterial territories. Collectively, these findings make paradoxical embolism less likely (9, 10). In addition, 24-hour Holter monitoring did not detect atrial fibrillation, making a cardioembolic mechanism less likely (11).

### 4.4 Therapeutic strategy and clinical decision-making

This case highlights the importance of multidisciplinary collaboration and individualized therapeutic decision-making in patients with complex thromboembolic disease. Given the immediate threat posed by pulmonary embolism, the multidisciplinary team prioritized pulmonary reperfusion therapy. Percutaneous pulmonary artery thrombectomy was successfully performed using the AcoStream aspiration catheter system, followed by standardized anticoagulation therapy with rivaroxaban. Recent studies have demonstrated that catheter-directed thrombectomy can rapidly reduce thrombus burden and improve hemodynamic parameters while avoiding the bleeding risks associated with systemic thrombolysis (12).

In contrast, although imaging demonstrated bilateral renal infarction, the patient's renal function remained within the normal range; collateral perfusion was maintained through bilateral accessory renal arteries; and the extent of renal infarction was relatively limited. Therefore, conservative management with anticoagulation therapy and close radiological follow-up was considered an appropriate strategy. Follow-up CTA demonstrated recanalization of the left renal artery and significant improvement of bilateral renal infarction lesions, supporting the effectiveness of this individualized treatment strategy. Moreover, the observed renal vascular recanalization and perfusion recovery after anticoagulation therapy are highly suggestive of renal artery embolism.

## 5 Conclusion

This case presents a rare coexistence of pulmonary embolism and bilateral renal artery embolism following LAVH. The patient achieved a successful outcome through individualized treatment, which was largely attributable to multidisciplinary collaboration. Furthermore, patient education regarding thromboembolic risk, strict adherence to anticoagulation, and early postoperative mobilization, together with proactive post-discharge surveillance, may be clinically important for improving long-term outcomes.

## Data Availability

The original contributions presented in the study are included in the article/Supplementary Material, further inquiries can be directed to the corresponding authors.
